# A preliminary study on oral health status and unmet dental needs in patients with home-based psychiatric services

**DOI:** 10.1038/s41598-026-35661-9

**Published:** 2026-01-09

**Authors:** Takayuki Suga, Trang Thi Huyen Tu, Yuji Gamo, Takafumi Asakura, Shigeru Iida, Yoko Iwase, Akira Toyofuku

**Affiliations:** 1 Department of Psychosomatic Dentistry, Graduate School of Medical and Dental Sciences, Institute of Science Tokyo, 1-5-45 Yushima, Bunkyo-ku, Tokyo, 113-8510 Japan; 2 Medical Corporation Group Keishi-kai, Tokyo, Japan; 3 Department of Basic Dental Sciences, Faculty of Dentistry, University of Medicine and Pharmacy at Ho Chi Minh City, Ho Chi Minh City, Vietnam; 4 Medical Corporation Group Sinsei-kai, Tokyo, Japan; 5 Medical Corporation Group Shinsui-kai, Tokyo, Japan; 6 Department of Dentistry for the Disability and Oral Health, Asahi University School of Dentistry, Gifu, Japan

**Keywords:** Oral health, Mental disorders, Home-based care, Unmet dental needs, Barriers to care, Medical-dental collaboration, Diseases, Health care, Medical research

## Abstract

To investigate the oral health status and unmet dental needs of patients receiving home-based psychiatric care in Tokyo. This preliminary study aimed to assess the extent of dental problems and identify the proportion of patients requiring urgent or comprehensive dental treatment to inform more effective integrated care strategies. This study involved 22 patients receiving psychiatric care at home from a Tokyo-based clinic. A single dentist conducted free, in-home dental examinations during psychiatric home visits. Oral health was evaluated using the decayed, missing, and filled teeth (DMFT) index, Oral Health Assessment Tool (OHAT), Oral Hygiene Index (OHI), Plaque Index (PI), and Tongue Coating Index (TCI). Patient demographic and clinical data were also analyzed. The findings revealed a high prevalence of untreated caries (elevated DT scores), residual roots, and poor oral hygiene across multiple indices. Many patients had not visited a dentist for several years. A significant majority (68.18%) of participants were recipients of public assistance, and financial constraints were identified as a critical barrier to accessing care for those ineligible. For 40.91% of participants, the examination was prompted by caregiver concern rather than patient initiative. Patients receiving home-based psychiatric care demonstrate significant unmet dental needs and poor oral hygiene, exacerbated by financial, motivational, and logistical barriers. The study highlights an urgent need for enhanced home dental services, improved financial support systems, and stronger collaboration among medical, dental, and social welfare sectors to provide continuous and accessible oral healthcare for this vulnerable population.

## Introduction

### Oral health status of patients receiving home-based psychiatric care

Patients with severe mental illnesses often suffer from poor oral health. Studies have found a high prevalence of dental caries and periodontal disease among individuals with psychiatric disorders, along with extensive unmet dental treatment needs^1^. For example, individuals with severe mental illness are nearly three times more likely to have lost all their teeth compared to the general population^2^. Contributing to this problem, many psychotropic medications have anticholinergic side effects that cause dry mouth, which in turn increases the risk of tooth decay and gum disease^3^. Moreover, symptoms of mental illness can hinder daily oral hygiene routines – research shows that people with serious mental illness engage in significantly fewer oral self-care behaviors (like regular toothbrushing) than others^4^. As a result of these factors, psychiatric patients frequently present with poor oral hygiene and untreated dental problems, such as decayed or broken teeth and infections^1^. Such findings are consistently echoed in the literature, underscoring that oral health is a major health disparity in this underserved group^5^.

### Dental care needs and barriers to utilization

Despite experiencing considerable dental morbidity, patients with chronic mental illness often receive only limited dental care. Dental attendance rates are notably low – one nationwide study found that only about 40% of patients with severe mental illness visited a dentist within a year, which is significantly below the rate in the general population^6^. This low utilization often results in oral health issues remaining unaddressed until they become emergencies. Multiple barriers contribute to the poor attendance. Economically, many patients face financial constraints or lack insurance coverage for comprehensive dental services. Psychologically, dental anxiety or phobia is common, and the prospect of a clinic visit can be overwhelming for individuals with a serious psychiatric condition. In addition, characteristics of mental illness (such as disorganization, cognitive impairment, or negative symptoms) may make it difficult for patients to schedule and keep a dental appointment. Indeed, a qualitative study reported that patients’ perceived barriers included limited access to dental care, fear of dental procedures, the effects of their illness on motivation, and lack of support for daily oral hygiene^7^. On the provider side, general dentists may feel ill-equipped to manage the special needs of psychiatric patients, and there is often a lack of formal referral pathways linking psychiatric services to dental care. Collectively, these patient-, provider-, and system-level barriers all contribute to the low rate of routine dental visits among people with mental health disorders^5,7^.

### Medical–dental collaboration

Individuals with psychiatric disorders frequently experience an oral health gap that stems in part from fragmented service delivery^8^. While mental health providers often concentrate on managing psychiatric symptoms, oral health concerns can remain unaddressed due to limited coordination among dental, psychiatric, medical services^9,10^. In many cases, this lack of integration perpetuates unmet dental needs and exacerbates existing health disparities among individuals with severe mental illness. Furthermore, for those receiving home-based psychiatric care, mobility constraints pose additional barriers to accessing routine dental treatment, as a significant number of these patients are homebound or find traveling to clinics particularly difficult^11^. Taken together, these factors underscore the complexity of oral health challenges faced by individuals with psychiatric disorders and highlight the importance of sustained collaboration between medical and dental professionals.

### Study objectives

In light of the aforementioned issues, this study aimed to elucidate the dental needs and challenges of patients receiving home-based psychiatric services. Free in-home dental examinations were conducted for individuals receiving regular home-based psychiatric care, and the findings were analyzed to assess their oral health status and dental treatment needs. The objectives of this research were (1) to assess the extent of dental problems in this population and (2) to identify the proportion of patients requiring urgent or comprehensive dental treatment. The ultimate aim is to generate evidence that can inform more effective integrated care strategies and improve oral health outcomes among individuals receiving home-based psychiatric care.

## Materials and methods

We approached 183 patients receiving home-based psychiatric care through the Itabashi Family Clinic, a psychiatric facility located in a residential area of Tokyo, to inform them about a free dental examination program. Furthermore, it is important to note that active recruitment was challenging for a subset of the initially approached individuals. For some, the severity of their psychiatric conditions limited interactions to brief doorstep conversations, precluding stronger recommendations for participation or rendering them unable to engage in the examination process. Of these patients, 24 (13.11%) expressed interest in the free dental examination. However, one patient declined participation on the day of the examination and another withdrew due to anxiety. Additionally, a few individuals who initially provided consent and did not necessarily have low daily living skills later withdrew; in these cases, the prospect of a novel interpersonal encounter with the dental examiner appeared to trigger past negative experiences, creating a psychological barrier to participation. This left 22 participants eligible for inclusion in this study. The free dental examinations were conducted from January 8 to February 6, 2025. A single dentist accompanied the psychiatric care team during home visits and performed the dental assessments immediately following the psychiatric consultation. Psychiatric care was provided by four different attending psychiatrists, whereas all dental examinations were performed by a single dentist.

### Ethical considerations

All procedures adhered to the principles of the Declaration of Helsinki and were reviewed and approved by the Dental Research Ethics Committee of Tokyo Medical and Dental University (approval number: S2025-003).

The informed-consent process proceeded in four coordinated steps. First, psychiatrists invited patients receiving home-based psychiatric care to attend a free dental examination; at registration for the examination, they explained that study participation was optional and obtained written, broad consent to participate from all registrants. Second, during the free dental examination itself, the dentist again provided a plain-language explanation of the study and reconfirmed each person’s willingness to participate. Third, because many registrants did not proceed to subsequent dental care after the free dental examination, the Ethics Committee approved the use of an opt-out procedure: the research protocol was posted on the clinic and the affiliated institution websites with a clearly indicated mechanism to withdraw consent. Finally, for participants who subsequently received treatment after the free dental examination, the dentist personally explained the study in non-technical language based on the protocol and obtained verbal consent prior to inclusion.

### Study variables

Patient demographic and clinical data were extracted from medical records, including age, gender, previous and current medical history (notably psychiatric diagnosis), living arrangement (e.g., whether living alone), current smoking status (yes/no), current psychotropic medication use, and receipt of welfare assistance such as public assistance. The diagnosis of psychiatric disorders was confirmed by the patients’ attending psychiatrist. Psychiatric disorders were classified according to the Diagnostic and Statistical Manual of Mental Disorders, Fifth Edition, Text Revision (DSM-5-TR).

During the free dental examinations, information was obtained regarding the chief oral complaint, the individual expressing concern about the patient’s oral condition, the date of the patient’s most recent dental visit, the current number of teeth (including wisdom teeth), the presence or absence of oral dyskinesia, the DMFT score, the Oral Health Assessment Tool (OHAT) score, the Oral Hygiene Index (OHI) score, the Plaque Index (PI), and the Tongue Coating Index (TCI). OHAT was originally developed as an oral-health screening instrument suitable for use by non-dental healthcare professionals. In the present study, OHAT was selected as the evaluation metric to facilitate seamless referral from non-dental healthcare providers to dental services. Based on the status of oral hygiene, the necessity for dental treatment and oral care was evaluated and explained to patients and their families, who were then asked whether they desired dental intervention^12–15^.

“DMFT” stands for decayed, missing, and filled teeth. In this study, DT refers to the number of teeth with untreated caries, MT refers to the number of teeth lost for any reason (excluding unerupted wisdom teeth), and FT refers to the number of teeth that have been treated for caries. The DMFT score is the sum of DT, MT, and FT.

### Statistics

The distributions of age and all scale scores were assessed for normality using the Shapiro–Wilk test. When the data followed a normal distribution or when comparison with previous studies was required, values were reported as the mean ± standard deviation (SD). If the distribution was normal but contained outliers, or if it was not normal, values were expressed as the median with interquartile range [IQR]). Statistical analysis was performed using IBM SPSS Statistics (version 29.0; IBM Corporation, Armonk, NY, USA).

### Sample size considerations

This investigation was designed as an exploratory, cross-sectional pilot study. The sample size was determined by feasibility and included all patients who consented to participate in the free in-home dental examinations during the study period (*n* = 22).

### Declaration of generative AI and AI-assisted technologies in the writing process

During the preparation of this study, the authors employed the Gemini 2.5 Pro (Google) system to translate the manuscript from Japanese into English, improve readability, rephrase text where appropriate, and ensure proper grammar. Subsequently, the authors conducted a thorough review and made any necessary editorial revisions. The authors assume full responsibility for the final content presented in this publication.

## Results

Table 1 presents the demographic and oral hygienic characteristics of individuals who participated in the free dental examinations. According to the TCI, 7 participants (31.82%) had scores of 9 or higher, indicating poor oral hygiene^16^. Table 2 summarizes the psychiatric diagnoses made by psychiatrists, with the majority of patients diagnosed with Schizophrenia Spectrum and Other Psychotic Disorders (10, 45.45%) or Depressive disorders (7, 31.82%). Table 3 shows the distribution of psychiatric diagnostic categories and current psychotropic medication use in the study sample.

**Table 1:** Patient background and oral hygiene status scores.

Variable (*n*=22)	
Age (yr)^a^	57.23±17.94
Gender (male/ female)	12 (54.55%)/10 (45.45%)
Living alone (Yes/No)	13 (59.09%)/9 (40.91%)
Concern origin: (Patient / Psychiatrist / Family)	13 (59.09%)/3 (13.64%)/6 (27.27%)
Public assistance recipient (Yes/No)	15 (68.18%)/7 (31.82%)
Current smoker (Yes/No)	4 (18.18%)/22(81.82%)
Oral dyskinesia (Present/Absent)	2 (9.09%)/20 (90.91%)
Months elapsed since last dental consultation^b^	24 (IQR 6–66)
Current tooth count (including wisdom teeth)^b^ Maximum: 32	26.5 (IQR 21–29)
DMFT score^b^ Maximum: 32	21 (IQR 18.5–25.5)
DT^b^	6 (IQR 4–15.5)
MT^b^	1.5 (IQR 0–7)
FT^b^	7.5 (IQR 3–11)
Oral health assessment tool (OHAT) score^a^ Maximum: 16	6.91±1.51
Oral hygiene index (OHI) score^a^ Maximum: 12	4.08±2.61
Debris index (DI)^a^	3.33±2.20
Calculus index (CI)^a^	0.74±0.96
Plaque index (PI) score^a^ Maximum: 3	1.68±0.95
Tongue coating index (TCI) score^a^ Maximum: 18	6.41±5.38
Number of patients needing dental consultation	22 (100%)
Dental intervention preference (Yes / No / Considering)	18 (81.82%)/2 (9.09%)/2 (9.09%)
Public assistance recipients among those seeking intervention	13 (72.22%)

^a^Values are presented as means (± standard deviation, SD), ^b^Median (Interquartile Range(IQR)).

**Table 2:** Psychiatric diagnoses (Including comorbidities).

Diagnosis	
Schizophrenia spectrum and other psychotic disorders	10
Depressive disorders	7
Neurocognitive disorders (Including one suspected case)	4
Neurodevelopmental disorders	3
Bipolar and related disorders	2
Sleep-wake disorders	2
Somatic symptom and related disorders	1
Feeding and eating disorders	1
Trauma- and stressor-related disorders	1
Anxiety disorders	1
Substance-related and addictive disorders	1

**Table 3:** Psychiatric diagnostic categories and psychotropic medication use among participants.

Patient number	Age (yr)	Gender	Diagnosis (DSM-5-TR–based classification)	Psychotropic medication use (/day)
1	53	Male	Neurodevelopmental disorders, depressive disorders	sodium valproate (fine granules 40%) 2 g, diazepam 4 mg, eszopiclone 2 mg, suvorexant 15 mg
2	43	Male	Schizophrenia spectrum and other psychotic disorders	haloperidol 3 mg, escitalopram 20 mg、alprazolam 0.8 mg、lemborexant 2.5 mg
3	38	Female	Anxiety disorders	quetiapine 100 mg, mirtazapine 45 mg, sodium valproate (fine granules 40%) 1 g, cloxazolam 2 mg, bezafibrate 400 mg
4	73	Female	Somatic symptom and related disorders, suspected neurocognitive disorders	quetiapine 100 mg, clonazepam 0.5 mg, lemborexant 2.5 mg
5	50	Male	Schizophrenia spectrum and other psychotic disorders, neurodevelopmental disorders, sleep-wake disorders, feeding and eating disorders	lurasidone 60 mg, trazodone 50 mg, guanfacine 6 mg, sodium valproate 1000 mg, topiramate 75 mg, carbamazepine 200 mg, clonazepam 0.5 mg, lemborexant 10 mg
6	53	Male	Schizophrenia spectrum and other psychotic disorders	perospirone 48 mg, brexpiprazole 2 mg, bromazepam 2 mg, suvorexant 20 mg, eszopiclone 3 mg
7	44	Female	Depressive disorders	sertraline 150 mg, flunitrazepam 2 mg, lorazepam 1 mg, suvorexant 20 mg
8	90	Male	Schizophrenia spectrum and other psychotic disorders, neurocognitive disorders	risperidone 1.5 mg, lemborexant 5 mg
9	63	Female	Depressive disorders, trauma- and stressor-related disorders	quetiapine 62.5 mg, duloxetine 60 mg, clonazepam 2 mg, lemborexant 2.5 mg
10	48	Male	Schizophrenia spectrum and other psychotic disorders, depressive disorders	paliperidone 6 mg, aripiprazole 12 mg, sodium valproate 600 mg, nitrazepam 10 mg, zolpidem 5 mg, lemborexant 2.5 mg
11	71	Female	Schizophrenia spectrum and other psychotic disorders	haloperidol 9 mg
12	27	Male	Bipolar and related disorders	sulpiride 50 mg, fluvoxamine 50 mg, sodium valproate 600 mg, bromazepam 2 mg
13	55	Female	Schizophrenia spectrum and other psychotic disorders	blonanserin (tape) 20 mg
14	76	Male	Schizophrenia spectrum and other psychotic disorders	quetiapine 75 mg
15	60	Female	Schizophrenia spectrum and other psychotic disorders	blonanserin (tape) 40 mg
16	63	Male	Schizophrenia spectrum and other psychotic disorders, neurodevelopmental disorders, neurocognitive disorders	lithium carbonate 200 mg, valproate (as sodium valproate) 800 mg, suvorexant 15 mg
17	88	Female	Depressive disorders	duloxetine 20 mg, lemborexant 5 mg
18	26	Male	Depressive disorders	mianserin 20 mg, bromazepam 10 mg, flunitrazepam 2 mg
19	80	Female	Depressive disorders	levomepromazine 20 mg, paroxetine 25 mg, flunitrazepam 2 mg, bromazepam 4 mg, zolpidem 10 mg
20	55	Male	Schizophrenia spectrum and other psychotic disorders	blonanserin 24 mg, diazepam 15 mg, lemborexant 10 mg
21	35	Female	Schizophrenia spectrum and other psychotic disorders	aripiprazole 3 mg
22	65	Male	Substance-related and addictive disorders, sleep-wake disorders	trazodone 50 mg, lemborexant 10 mg

DSM-5-TR: The Diagnostic and Statistical Manual of Mental Disorders, Fifth Edition, Text Revision.

**Fig. 1:**
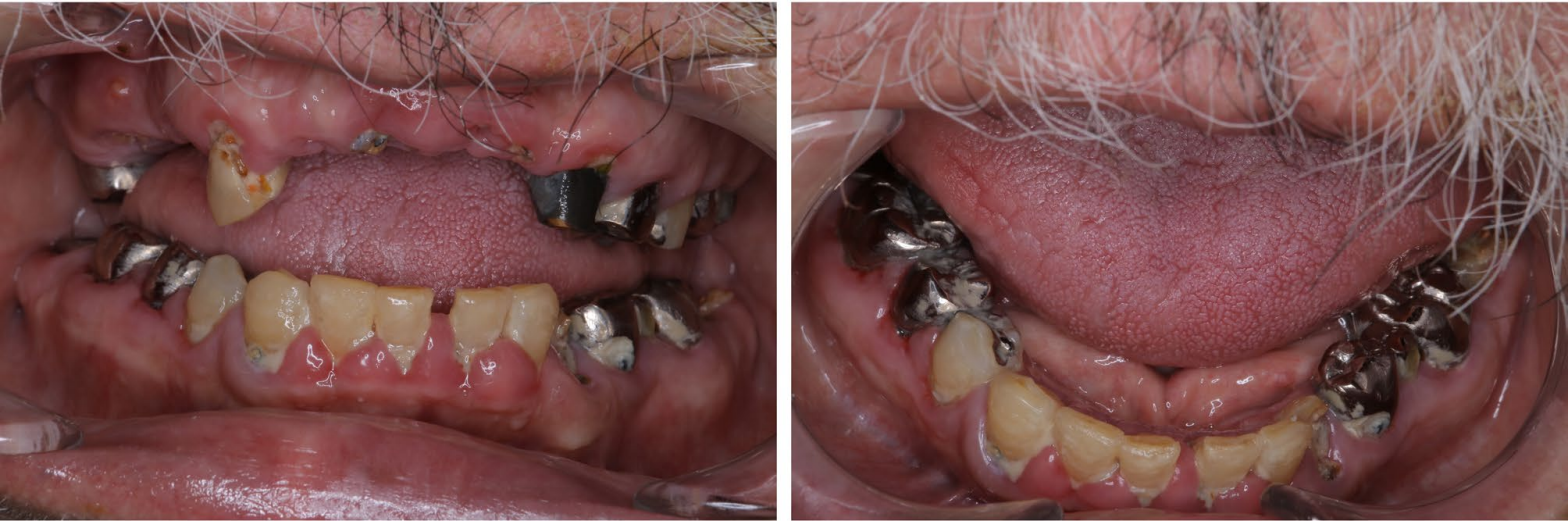
The images presented in Fig. 1 depict the intraoral condition of a participant in the free dental examinations. This individual was diagnosed with schizophrenia and reported not engaging in any tooth brushing. Numerous untreated carious lesions and residual roots are evident, and the anterior mandibular region shows gingival hyperplasia, presumably associated with the use of antihypertensive medication. Notable plaque accumulation is present across the dentition, and calculus can be observed on the occlusal surfaces of the lower right molars—a location where calculus is typically uncommon.

Figure 1 shows intraoral photographs of a representative participant from the free dental examinations. This patient had a diagnosis of schizophrenia and reported not engaging in regular tooth brushing. Multiple carious lesions requiring dental treatment and residual roots were observed, and the mandibular anterior region displayed gingival hyperplasia, presumably due to the side effects of antihypertensive medications. In addition, widespread plaque accumulation was noted, and calculus was observed on the occlusal surfaces of the lower right molars—a location where calculus is not typically found.

## Discussion

### Oral health status

In this study, the current tooth count was 26.5 (IQR 21–29), which is nearly equivalent to the reported number of 26.5 teeth for the general Japanese population aged 55–59^17^. In contrast, although direct comparison with a previous study is limited, the DMFT score was higher than that reported for psychiatric inpatients at a hospital in Tokyo^18^. Component analysis revealed a higher DT score and a lower FT score. In the present study, we observed numerous residual roots, which are the remnants of teeth severely decayed to the point of requiring extraction. This clinical finding suggests that necessary treatments, such as extractions, were not performed due to patients’ long-term avoidance of dental visits. This avoidance of professional care, combined with limited self-care and insufficient support from families and healthcare providers, allowed dental caries to progress untreated to a severe state^19^. During the free dental examinations we frequently observed large stocks of sugar‑sweetened beverages (SSBs) in the participants’ homes. This field impression mirrors the community‑based study by Lambert et al., who quantified a mean 86 g/day of added sugars from SSBs in adults with severe mental illness—nearly four‑fold the intake of the general population^20^. Such readily fermentable sugars provide continuous substrate for Streptococcus mutans, accelerating the rampant caries and elevated DT values documented in our cohort. Therefore, interventions that curb household availability of SSBs should accompany any strategy aimed at improving oral self‑care in home‑bound psychiatric patients.

Furthermore, the results indicated that a considerable number of patients had not visited a dentist for several years, and a high proportion were assessed as having poor oral hygiene based on multiple indices, including OHAT, OHI, PI, and TCI. Specifically, the OHAT score was 6.91 ± 1.51, which exceeds the value of 4.2 ± 2.6 reported for Japanese elderly home-care patients (mean age 84.0 ± 7.8) who receive visiting nurse services^21^. The OHI score was 4.08 ± 2.61, higher than the 2.83 ± 1.54 reported for Japanese patients with pneumonia (mean age 73.9 ± 13.1) in Japan^22^. The PI score was 1.68 ± 0.95, surpassing the 0.91 ± 0.62 reported for patients (mean age 43.21 ± 13.24) at the periodontology department of a Brazilian university hospital^23^. In addition, 31.82% of patients were classified as having poor oral hygiene according to the TCI. This percentage was higher than the 15.79% reported in an Indonesian study of older adults^24^.

These findings collectively indicate that patients in this study had poor overall oral hygiene. Contributing factors may include a characteristic tendency among individuals with psychiatric disorders to avoid dental treatment, insufficient self-care, and inadequate support from caregivers (family and medical staff). Furthermore, the high DT score may reflect the persistence of untreated caries and the avoidance of extractions, leading to sustained high levels of decayed teeth. Notably, there is the presence of multiple untreated teeth left only as residual roots, which not only increases the risk of infection but may also compromise masticatory function and overall nutritional status.

Psychotropic medication use is another important factor to consider when interpreting the present findings. Many antipsychotic, antidepressant, and anxiolytic agents have anticholinergic properties that reduce salivary flow and cause xerostomia, thereby increasing susceptibility to dental caries, periodontal disease, and oral infections. In the present sample, a range of psychotropic regimens was observed (Table 3), and this medication-related xerostomic burden may have contributed to the high prevalence of oral health problems identified in this population. Future studies with larger samples and more detailed information on individual drug classes, dosages, and treatment duration will be required to clarify the specific relationships between psychotropic medication profiles, xerostomia, and oral health outcomes.

In conclusion, improving oral hygiene status is an urgent priority for individuals with psychiatric disorders receiving home-based psychiatric care. Regular dental visits and comprehensive self-care instruction should be strongly promoted to address these oral health concerns.

### Caregiver involvement and continuous support

Individuals with psychiatric disorders frequently exhibit reduced self-management skills and low motivation, resulting in the neglect of daily oral care^25^. In particular, individuals with psychiatric disorders often struggle to manage their overall health. This challenge is further compounded by psychological barriers to dental treatment (e.g., fear or anxiety related to dental care, difficulty leaving home), making basic oral hygienic practices like tooth brushing more likely to be neglected. In the present study, 40.91% of participants sought dental care primarily because their attending psychiatrist or family members had expressed concern about their oral condition. While this reflects proactive involvement by caregivers in monitoring patients’ oral health status, it also underscores the possibility that patients themselves are not sufficiently conscious of or committed to oral care.

Moreover, the fact that oral hygiene conditions often deteriorate further despite their seriousness suggests a lack of shared awareness regarding oral care between patients and caregivers, and it also highlights the potential risks when caregiver support is insufficient. The importance of robust support systems, encompassing both familial involvement and financial assistance, in facilitating access to continued dental care was clearly demonstrated by follow-up data. As of June 2025, seven participants had progressed from the free dental examination to actual home-visit dental treatment. Notably, six of these seven individuals either lived with family members or had family present during dental treatments, indicating strong familial support. Furthermore, all seven patients incurred no out-of-pocket expenses for the home-visit dental services, as five were recipients of public assistance and two were receiving disability welfare benefits that covered their treatment costs. Echoing these observations, the strong commitment of caregivers sometimes facilitated dental service access even for patients with severe psychiatric symptoms whose ability to provide explicit consent was compromised or who initially seemed unlikely to engage. In some cases, the proactive stance of family members appeared to be a decisive factor in overcoming patient-related reluctance and connecting them to care, raising considerations about the role of supportive decision-making or a form of protective medical intervention driven by caregivers. Multiple factors—including limited encouragement from caregivers to seek dental care and avoidance of dental care by the patients themselves—interact to create conditions under which oral hygiene fails to improve as expected^7^. However, when caregivers actively provide routine support, such as supervising tooth brushing and monitoring oral conditions, there is considerable potential to improve and maintain patients’ oral hygiene. In situations where patients with mental disorders find it difficult to perform oral care independently, caregivers must provide appropriate interventions within a well-structured support framework.

Strengthening the role of caregivers requires the development of educational programs and support systems. For instance, workshops for families and other supporters that convey the correct methods of oral care and emphasize its importance, as well as collaborative support systems facilitated by visiting nurses and physicians (including psychiatrists), can be highly effective^26^. Clearly defining the type and scope of support that caregivers can provide may improve the quality of patients’ oral hygiene management, ultimately promoting better oral health maintenance and overall well-being.

Looking ahead, further research should focus on how to establish caregiver support structures and educational programs, as well as on the specific methods of providing ongoing support to patients. Moreover, as collaboration with caregivers advances, monitoring changes in the continuity and improvement of patients’ oral care, and evaluating the effectiveness of these changes, will be critical research tasks in the future.

### Appropriate dental treatment according to the patient’s condition

To the best of our knowledge, no previous studies have specifically addressed dental treatment or oral care among home-based individuals with psychiatric disorders who receive visiting dental services. Considering factors such as stress associated with dental procedures, limited understanding of dental treatment objectives, restricted mouth opening, and impaired swallowing and masticatory function, it is considered difficult to provide conventional dental treatment in a standard dental clinic setting. Indeed, the sobering reality is that standard dental procedures are difficult to perform in these patients, and improving oral hygiene alone is unlikely to ameliorate their underlying psychiatric conditions.

Atraumatic restorative treatment (ART) is a minimally invasive caries management method involving hand excavation of demineralized dentin and placement of a high-viscosity glass ionomer filling^27^. It preserves healthy tooth structure, reduces patient discomfort (often eliminating the need for anesthesia), and is especially advantageous when adequate moisture control is difficult and conventional composite resin restorations are therefore impractical^28^. In addition, glass ionomer filling has a fluoride releasing property, which provides a higher level of caries prevention compared to composite resin restorations^29^. One study reported that ART is effective in vulnerable groups such as older adults^30^.

Although not specifically involving individuals with psychiatric disorders, Genaro et al. reported that in home-based dental care for older adults, approximately 74% of restorations using ART remained in good condition after one year, suggesting its potential as an effective dental treatment method^31^. In individuals with psychiatric disorders, particularly those receiving home-based dental care, maintaining prolonged mouth opening can be difficult, and the risk of accidental ingestion or aspiration is elevated. Moreover, achieving adequate moisture control—crucial in conventional dental treatments—is difficult, and poor oral self-care frequently contributes to recurrent caries. Therefore, ART may represent a suitable alternative. Future research should investigate the application of ART in visiting dental care for psychiatric patients to demonstrate its efficacy.

### High rate of public assistance recipients and the existence of patients unable to receive dental treatment

Based on the results of this study, 68.18% of the participants were receiving public assistance. This proportion is substantially higher than the 2023 public assistance rate in Tokyo, which was 9.8 per 1000 population, as reported by the Tokyo Metropolitan Government Bureau of Social Welfare^32^. Even considering the vulnerability inherent in a population with mental disorders, the fact that the rate of public assistance recipients is remarkably high is a noteworthy characteristic.

Under the Japanese healthcare system, patients receiving public assistance are able to receive insured medical services—including home psychiatric and dental care—at no cost. However, patients not covered by public assistance are required to pay a copayment of approximately 2000–6000 yen per home dental visit (calculated 14–40 USD at 150 yen /USD), depending on age and income. For unemployed individuals receiving home psychiatric care, even this amount can pose a substantial financial burden. In this study, 81.82% of those who underwent the free oral examination subsequently expressed a desire for further dental treatment or oral care. However, financial considerations emerged as a barrier for some. For instance, one participant not receiving public assistance ultimately declined treatment due to cost, and another postponed care for the same reason. The profound impact of financial barriers was further highlighted by anecdotal evidence from the broader group approached for the study, where some individuals reportedly delayed necessary dental treatment until they secured public assistance, as their dwindling financial resources prevented them from affording care. This reinforces the notion that out-of-pocket costs, or the lack of resources to cover them, represent a critical barrier to accessing dental services for this vulnerable population, even preceding considerations of public assistance eligibility. Separately, other participants in this study who also were not public assistance recipients but did proceed with and receive dental treatment were found, during subsequent home dental visits, to have been certified as disabled due to mental illness. Consequently, these individuals received support from other welfare programs, which covered their dental care costs, resulting in no out-of-pocket expenses for them. While this outcome demonstrates that alternative financial support pathways can exist for some, the initial declination and postponement of care by others due to cost concerns underscore that financial constraints may indeed limit access to oral care, particularly for those not readily aware of or eligible for such alternative welfare systems.

In the future, a comprehensive support system—encompassing medical and welfare collaboration—will be necessary in view of these healthcare cost burdens and the unique difficulties that individuals with psychiatric disorders face in accessing care. In particular, it will be important to strengthen the coordination between home dental care and home psychiatric care so that appropriate oral care and psychiatric services are accessible regardless of public assistance status. By considering measures to reduce economic barriers and enhancing communication among healthcare professionals, it is anticipated that continued oral hygiene management can be provided even to patients in vulnerable circumstances.

### Policy considerations

First, to support individuals with high medical needs and significant financial hardship—including those receiving home-based psychiatric care—it is advisable to expand the existing subsidy system for home-visit dental services must be expanded. For low-income individuals or patients with severe mental illness who are not eligible for public assistance, a supplemental public funding mechanism beyond standard health insurance coverage should be introduced. This would help eliminate financial barriers to accessing home dental care and promote equitable service delivery. Concretely, drawing on the model of medical assistance provided to individuals with disabilities or specified diseases, the introduction or expansion of dedicated financial support for home dental services emerges as a critical step towards addressing the needs of patients with mental disorders. These services should be structured to ensure ease of use, broad accessibility, and continuity of care, thereby facilitating appropriate utilization among individuals who may otherwise face significant barriers.

Second, strengthening collaboration among medical, dental, and welfare services is vital to establish a system that does not overlook dental care needs. During home visits by psychiatrists or nurses, it is crucial to conduct an oral health assessment or basic screening. When issues are identified, a prompt and reliable referral pathway to home dental services must be available. Simultaneously, legal frameworks and IT infrastructures should be developed to allow information sharing among municipalities, public health centers, and community comprehensive support centers. This will help realize a “continuous medical support system” without gaps in care.

Furthermore, it would be worthwhile to establish a system that supports “regular free dental examinations” and “preventive interventions.” As observed in the present study, once patients receive a free dental examination, many become motivated to seek further dental treatment. Accordingly, municipalities or home-visit care teams should regularly organize opportunities for oral health assessments. If dental issues are found during screening, a structured mechanism should be in place to ensure timely connection with appropriate dental treatment and oral care specialists. These measures could not only enhance the quality of life (QOL) for individuals with mental disorders, but also promote more efficient allocation of healthcare expenditures.

Through such integrated efforts, the “disparities in access to dental care” can be minimized, and it is anticipated that society can move closer to a state in which all individuals—including those with mental disorders—can consistently receive the dental services necessary to maintain optimal oral health.

### Future studies

Building on these considerations, future research should encompass cohort studies with larger sample sizes and the evaluation of specific interventions—such as restorative procedures using ART and effective oral care programs. To determine the most effective approaches, it will be essential to employ study designs that integrate multifaceted assessments, including patients’ mental health status and medication profiles.

### Limitation

#### Limited sample size

This study included only 22 participants, thereby restricting the generalizability of its findings. Moreover, the small sample size made it challenging to conduct detailed analyses and comparisons across subgroups (e.g., mental disorder types, social backgrounds). Because no a priori sample size calculation was performed, we conducted a sensitivity power analysis based on the final sample size. With a two-sided α of 0.05 and 80% power, the study could reliably detect only moderate-to-large standardized mean differences (Cohen’s d ≈ 0.60); smaller differences may therefore have gone undetected. Larger, adequately powered multicenter studies are warranted to confirm these findings and examine more subtle effects.

#### Limited generalizability due to a single-center design

The investigation targeted patients from a single psychiatric home-visit clinic, which may not reflect variations in regional factors, healthcare institutions, or support systems. Consequently, caution is advised when attempting to extrapolate these results to other regions or healthcare settings.

#### Selection bias arising from targeting individuals interested in a free dental examinations

This study was limited to individuals who voluntarily requested a free dental examination, raising the possibility that the sample predominantly included individuals already motivated or concerned about dental care. Consequently, the findings may not fully capture the oral care status and healthcare-seeking behaviors of the broader home-based psychiatric patient population.

Moreover, it is possible that patients with more severe mental illness may have had little to no interest in the free dental check-up from the outset, resulting in their underrepresentation in the study sample.

## Conclusion

In this study, free dental examinations were provided for patients receiving home-based psychiatric care. All patients were found to have untreated dental caries and inadequate oral hygiene. These findings indicate that the features commonly associated with mental disorders—including impaired self-care motivation, dental anxiety, and psychological or physical barriers to leaving the home—are major contributors to reduced access to dental care, particularly when compounded by financial hardship. In particular, a patient who does not receive public welfare benefits reported having to discontinue dental treatment due to the costs associated with home dental visits, indicating that the presence or absence of financial support may critically influence access to dental services. Based on these observations, the need for enhanced home dental services, improved financial support, and the establishment of a continuous care system through collaboration among medical, dental, and social welfare sectors is evident. Addressing these issues is crucial for improving the oral health of patients with mental disorders and represents a significant challenge to be met.

## Data Availability

The datasets generated and analyzed during the current study are not publicly available due to privacy and confidentiality agreements but are available from the corresponding author on reasonable request.
